# Arterial Thromboembolism in Patients With Advanced Lung Cancer: Secondary Analyses of the Rising‐VTE/NEJ037 Study

**DOI:** 10.1002/cam4.70568

**Published:** 2025-01-09

**Authors:** Naoki Furuya, Yukari Tsubata, Takamasa Hotta, Toshihide Yokoyama, Masahiro Yamasaki, Nobuhisa Ishikawa, Kazunori Fujitaka, Tetsuya Kubota, Kunihiko Kobayashi, Takeshi Isobe

**Affiliations:** ^1^ Division of Respiratory Medicine, Department of Internal Medicine St. Marianna University School of Medicine Kawasaki Japan; ^2^ Division of Medical Oncology and Respiratory Medicine, Department of Internal Medicine Shimane University Faculty of Medicine Izumo Japan; ^3^ Department of Respiratory Medicine Kurashiki Central Hospital Kurashiki Japan; ^4^ Department of Respiratory Disease Hiroshima Red Cross Hospital and Atomic‐Bomb Survivors Hospital Hiroshima Japan; ^5^ Department of Respiratory Medicine Hiroshima Prefectural Hospital Hiroshima Japan; ^6^ Department of Respiratory Medicine Hiroshima University Hospital Hiroshima Japan; ^7^ Department of Respiratory Medicine and Allergology Kochi University Hospital Kochi Japan; ^8^ Department of Respiratory Medicine Saitama Medical University International Medical Center Saitama Japan

**Keywords:** arterial thromboembolism, cancer‐associated thromboembolism, direct oral anticoagulants, lung cancer, prospective cohort study

## Abstract

**Background:**

Cancer‐associated thromboembolism has been thoroughly investigated in previous studies, and direct oral anticoagulants (DOACs) were established for the treatment and prevention of venous thromboembolism (VTE). However, the risks of cancer‐associated arterial thromboembolism (ATE) and the efficacy of DOACs remain unclear.

**Objectives:**

To evaluate the risk factors and the clinical activity of edoxaban (EDO) for the prevention of ATE in patients with advanced lung cancer.

**Methods:**

From the prospective Rising‐VTE/NEJ037 study which investigated VTE in newly diagnosed advanced lung cancer, we investigated the incidence rate and the risk factors of ATE as secondary endpoints.

**Results:**

A total of 1008 patients were screened for VTE at study baseline and were followed up for 2 years. Excluding patients with a contraindication to DOACs, those with VTE were treated with EDO. ATE events were identified in 41 patients (4.1%). The most common location for ATE was cerebral infarction (*N* = 31, 75.6%), followed by myocardial infarction (*N* = 4, 9.8%). Multivariate analysis determined the incidence of VTE, D‐dimer, a comorbidity of atrial fibrillation, and four other factors as independent risk factors of ATE. For VTE (+) patients, the incidence rate of ATE was 15.9% for the EDO administration (+) patients, compared with 11.1% for the EDO administration (−) patients (*p* = 0.626).

**Conclusions:**

The incidence rate of ATE was 4.1% over 2‐year follow‐up in advanced lung cancer patients. VTE was further identified as an independent risk factor for ATE, while intervention with DOACs was seen as less effective for the prevention of ATE in advanced lung cancer patients with VTE.

**Trial Registration:**

This trial was registered in the Japan Registry of Clinical Trials (jRCTs061180025)

## Graphical abstract

The incidence rate of arterial thromboembolism (ATE) was 4.1% over a 2‐year follow‐up in advanced lung cancer patients. Venous thromboembolism (VTE) was further identified as an independent risk factor for ATE, while intervention with direct oral anticoagulants was seen as less effective for the prevention of ATE in advanced lung cancer patients with VTE.

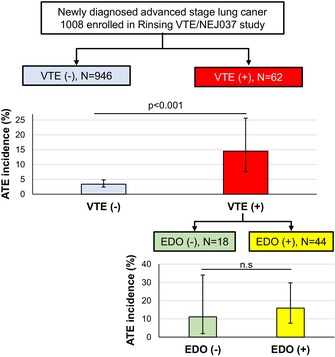

## 1 Background

Cancer‐associated thromboembolism (CAT) is a critical comorbidity for cancer patients [1, 2, 3]. Cancer‐associated hypercoagulability leads to both venous thrombo‐embolisms (VTE) and arterial thromboembolisms (ATE). Many previous studies that investigated CAT focused on the incidence rate, risk factors of VTE, and risk factors of bleeding [4, 5, 6, 7, 8]. Some of these risk factors were cancer‐related factors (cancer types [9, 10], genetic characteristics [11, 12, 13]) and other patient‐related factors [14, 15]. A large case–control study revealed that patients with hematological malignancies had the highest risk of VTE (adjusted odds risk (OR): 28.0), followed by lung cancer (adjusted OR: 22.2) and gastrointestinal cancer (adjusted OR: 20.3), compared to patients with‐out malignancies [9]. KRAS mutation‐positive non‐small cell lung cancer (NSCLC) was associated with an increased risk of VTE (OR: 2.67) compared to NSCLC patients without KRAS mutation [12], and ROS1 or ALK fusion‐positive NSCLC had a higher incidence of thromboembolisms than in EGFR mutated NSCLC [13].

Over the past 5 years, some pivotal randomized control trials revealed that direct oral anticoagulants (DOACs) were effective for the treatment and prevention of cancer‐associated VTE [16, 17, 18]. To date, we conducted the largest prospective cohort study, the Rising‐VTE/NEJ037, and have reported the incidence rate and the risk factors of VTE in patients with advanced lung cancer [19, 20]. The incidence rate of VTE, which was the primary endpoint of this study, was 9.9%. Moreover, we revealed that edoxaban (EDO) was highly effective in preventing VTE recurrence in lung patients with VTE [21].

However, as most of previous studies focused on VTE, the risk factors of ATE and the efficacy of DOACs for the prevention of cancer‐associated ATE have not been thoroughly investigated [3, 22]. ATE often leads to sudden symptomatic events and more critical comorbidity when compared to VTE in cancer patients [23]. The aim of this study was to reveal the risk factors and evaluate the clinical activity of EDO for the prevention of ATE in advanced lung cancer as secondary endpoints to the Rising‐VTE/NEJ037 study.

## 2 Methods

### 2.1 Study Participants

Patients were enrolled from June 2016 to August 2018 across 35 institutions in Japan. Eligible patients were (i) newly pathologically confirmed with non‐small cell lung cancer (NSCLC) or small cell lung cancer (SCLC), (ii) aged ≥ 20 years old, (iii) an Eastern Cooperative Oncology Group (ECOG) performance status (PS) of 0–3, (iv) unable to undergo radical surgery, definitive radiotherapy/chemoradiotherapy (regardless of TNM stage), and (v) a predictive prognosis > 6 months after consent. At each institution, eligible patients were consecutively enrolled. Patients were excluded if they were administrated with aspirin or other antiplatelet drugs at the study baseline. This study was conducted in accordance with the Helsinki Declaration and the Good Clinical Practice Guidelines. The study protocol was approved by the institutional review board at Shimane University (approval number: CRB6180008), and written informed consent was obtained from all patients before enrollment in this study. This trial was registered in the Japan Registry of Clinical Trials (jRCTs061180025).

### 2.2 Study Design and Endpoints

Rising‐VTE is a multicenter, prospective, observational cohort study. We aimed to identify the incidence rate and the risk factors of VTE in unresectable advanced‐stage lung cancer patients. Co‐primary endpoints were the incidence rate of VTE over the 2‐year study period and the VTE recurrence rate over 6 months after initiation of EDO. For patients with VTE (+) on baseline screening, investigators treated the patients with low molecular weight heparin (LMWH) before edoxaban (EDO). The initial dose of EDO was 60 mg daily oral administration (BW ≧ 60 kg). Secondary endpoints were the incidence rates of ATE, bleeding events, and overall survival.

**FIGURE 1:**
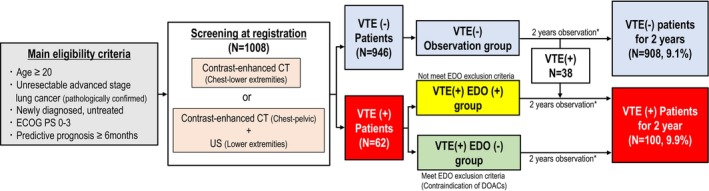
Study schema of Rising VTE/NEJ037 study. ECOG PS, Eastern Cooperative Oncology Group Performance Status; EDO, edoxaban; US, ultrasound sonography; VTE, venous thromboembolism. *After baseline screening of VTE, VTE/ATE screening was continued as clinical practice by the attending physician.

All enrolled patients underwent VTE screening by contrast‐enhanced CT scans from the chest to lower extremities (whole‐body CT) at lung cancer diagnosis, before initial anticancer treatment. A combination of chest to pelvic enhanced CT and lower extremity ultrasonography were also permitted in lieu of whole‐body enhanced CT. In the patients identified with VTE and receiving EDO at the baseline screening were defined as VTE (+)/EDO (+) group. On the other hand, the patients identified with VTE but were unable to undergo EDO administration were defined as VTE (+)/EDO (−) group (observation group). A detailed exclusion criteria for EDO administration were described in our previous article [20]. Based on the results of baseline VTE screening and EDO administration, were allocated into three groups, the VTE (−) observation group, VTE (+)/EDO (+) group, and VTE (+)/EDO (−) group (Figure 1). After the baseline screening, the patients underwent standard lung cancer treatment, and CT/MRI evaluations were continued by their attending physician over the 2‐year follow‐up as regular clinical practice. VTE/ATE events were defined by radiological confirmation with CT or US imaging by physicians. After 2‐year follow‐up period, VTE/ATE events were eventually confirmed by the independent review committee (IRC), which included two radiologists.

### 2.3 Definition of Arterial Thromboembolism

The definition of VTE was described in our previous article (11). ATE events included newly diagnosed cerebral infarction, myocardial infarction, and any ATE, which were confirmed by attending physicians and IRC.

### 2.4 Statistical Analysis

The Rising‐VTE study was aimed to identify the incidence rate of VTE and its risk factors. When planning this study, the incidence rate of VTE was unclear in advanced lung cancer patients. Thus the sample size of this study was aimed to exceed that of previous prospective cohort studies. As the previous studies included cases of the hundreds, the target sample size of this study was set to 1000 cases. The secondary endpoint of identification of ATE risk factors was performed by univariate and multivariate Fine‐Gray model analysis with death as a competing risk. First, we performed univariate by using the Fine‐Gray model for each factor. The outcome was defined as the onset of ATE, and the zero point was the date of study registration. We checked the proportional hazard nature of the main factor by using a double log plot, the presence or absence of VTE versus ATE. Next, we adopted multivariate analysis using a stepwise method to identify significant risk factors of ATE. Variables that we used in univariate analysis were age, sex, histology of lung cancer, clinical stage, ECOG PS, comorbidities (COPD, diabetes, hypertension, hyperlipidemia, rheumatoid arthritis, atrial fibrillation, other malignancies), medical history (cerebral infarction, myocardial infarction, other malignancies), anticoagulation status, hematological examination, SpO_2_, blood pressure, and incidences of VTE. Variables that were significant in univariate analysis were employed in the multivariate model. Variables with multicollinearity problems were excluded in a prior correlation analysis. Statistical analyses were performed using SPSS software, version 24.0 (IBM Japan., Tokyo, Japan).

## 3 Results

### 3.1 Baseline Patient Characteristics

**FIGURE 2:**
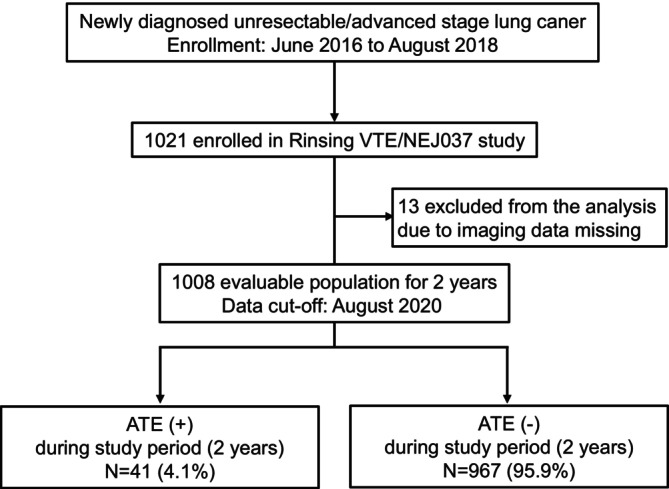
Study diagram of Rising VET/NEJ037 study for secondary endpoints of ATE incidence. ATE, arterial thromboembolism; VTE, venous thromboembolism.

Of the 1021 patients enrolled in this study, 13 patients were excluded due to imaging data missing. Finally, a total of 1008 cases were followed up as a full analysis set (Figure 2).

Baseline characteristics of patients are summarized in Table 1. Disease stage was assessed according to the 7th edition of the TNM staging system for lung cancer (UICC‐7), with M1a and M1b stage IV disease accounting for over 80% of patients.

**TABLE 1:** Patient characteristics at the time of lung cancer diagnosis (*N* = 1008).

	Evaluable population *N* = 1008 (100%)
Age (years)	
Median (range, IQR)	70 (30–94, 65–76)
Sex	
Male/Female	714 (70.8%)/294 (29.2%)
ECOG PS	
0/1/2/3	403 (40.0%)/490 (40.6%)/74 (7.3%)/41 (4.1%)
Histology of lung cancer	
Ad/Sq/Small/Others	641 (63.6%)/187 (18.6%)/137 (13.6%)/43 (4.3%)
Oncogenic driver gene aberration	
*EGFR* mutation	280 (27.8%)
*ALK* fusion	34 (3.4%)
*EGFR*/*ALK* (−)	480 (47.6%)
Unknown	214 (21.2%)
M factor of TNM classification	
M0/M1a/M1b	192 (20.0%)/228 (23.8%)/540 (56.3%)
TNM classification	
I/II/III/IV/Postoperative recurrence	2 (0.2%)/8 (0.9%)/101 (11.6%)/623 (71.5%)/137 (15.2%)
Anticancer treatment after registration	
(+)/(−)/Unknown	919 (91.2%)/84 (8.3%)/5 (0.5%)
Anticoagulation therapy	
(+)/(−)	59 (5.9%)/949 (94.1%)
Incidence of VTE (Primary endpoint)	
(+)/(−)	100 (9.9%)/908 (90.1%)

Abbreviations: Ad, adenocarcinoma; IQR, interquartile range; PS, performance status; Small, small cell carcinoma; Sq, squamous cell carcinoma; VTE, venous thromboembolism.

### 3.2 Incidence of ATE and Its Characteristics

During the study period, ATE events developed in 41 patients (*N* = 41, 4.1%, Figure 1). Baseline characteristics of patients who developed ATE are summarized in Table 2. These patients were male dominant (*N* = 30, 73.2%) with good PS, and a median age of 71 years (range: 47–87 years) at baseline screening. The most common histological subtype of lung cancer was adenocarcinoma (*N* = 25, 61.0%), followed by squamous cell carcinoma (*N* = 11, 26.8%). Over 90% of patients received anticancer treatment during the duration of the study. A history of cerebral infarction and myocardial infarction were seen in six patients and two patients, respectively. Common comorbidities were hypertension, COPD, diabetes, hyperlipidemia, and atrial fibrillation. In some patients, there was an overlap of multiple comorbidities. Approximately 30% of ATE (+) patients further developed VTE during the observation period (*N* = 12, 29.3%).

**TABLE 2:** Patient characteristics of ATE‐developed patients (*N* = 41).

	ATE developed patients *N* = 41 (Incidence rate: 4.1%)
Age (years)	
Median (range, IQR)	71 (47–87, 63–75)
Sex	
Male/Female	30 (73.2%)/11 (26.8%)
ECOG PS (at the study registration)	
0/1/2/3	10 (24.4%)/26 (63.4%)/2 (4.9%)/3 (7.3%)
Histology of lung cancer	
Ad/Sq/Small/Others	25 (61.0%)/11 (26.8%)/4 (9.8%)/1 (2.4%)
Anticancer treatment (after registration)	
(+)/(−)	37 (90.2%)/4 (9.8%)
Anticoagulation status	
(+)/(−)	7 (17.1%)/34 (82.9%)
Medical history	
Cerebral infarction	6 (14.6%)
Myocardial infarction	2 (4.9%)
Renal infarction	1 (2.4)
Comorbidity^a^	
Hypertension	16 (39.0%)
COPD	13 (31.7%)
Diabetes	8 (19.5%)
Hyperlipidemia	8 (19.5%)
Atrial fibrillation	6 (14.6%)

Abbreviations: Ad, adenocarcinoma; ATE, arterial thromboembolism; IQR, Interquartile range; PS, performance status; Small, small cell carcinoma; Sq, squamous cell carcinoma.

^a^ Multiple comorbidities were overlapped in some patients.

### 3.3 Location of ATE

**FIGURE 3:**
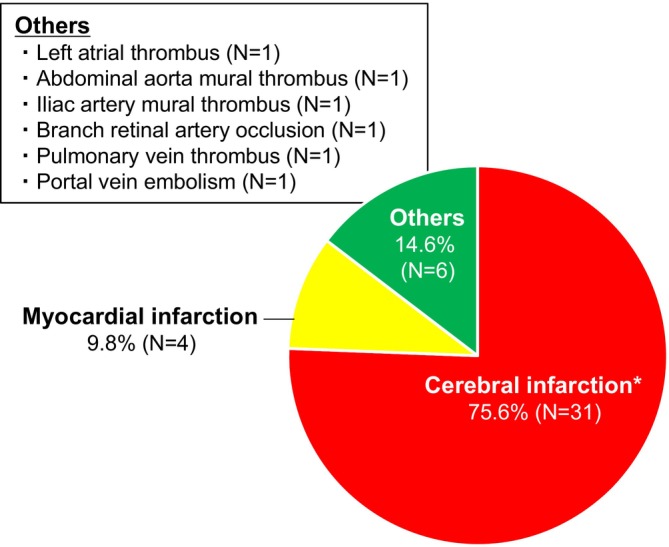
Location of ATE (*N* = 41). *One patient co‐developed splenic infarction. ATE, arterial thromboembolism.

Three‐quarters of all ATE events were cerebral infarction (*N* = 31, 75.6%). One patient co‐developed cerebral infarction and splenic infarction. Of the 31 patients who developed cerebral infarction, five patients had a history of cerebral infarction at baseline. Myocardial infarction developed in four patients (9.8%). Further details of ATE locations are shown in Figure 3.

### 3.4 Risk Factors for ATE Development

Since confirmation of the double log plot showed no significant loss of proportional hazards, we performed an association analysis with the Fine‐Gray model. After the univariate analysis, including the patient background data, tumor‐related factors, physiological examinations, and hematological examination results, multivariate analysis identified five factors (incidence of VTE, neutrophil, D‐dimmer, ALT, and atrial fibrillation) as risk factors for ATE (Table 3). In particular, the incidence of VTE, D‐dimer, and comorbidity of atrial fibrillation were strong risk factors for the development of ATE (*p* < 0.001).

**TABLE 3:** Risk factors for ATE by the Fine‐Gray model.

Variables		Univariate analysis		Multivariate analysis	
		HR (95% CI)	*p* value	HR (95% CI)	*p* value
VTE (+)	vs. (−)	4.08 (2.08–7.99)	< 0.001	3.25 (1.66–6.36)	< 0.001
Medical history of cerebral infarction (+)	vs. (−)	2.65 (1.10–6.38)	0.03	Not selected	
Neutrophil	Per 1%	1.06 (1.02–1.09)	0.001	1.04 (1.01–1.08)	0.008
PT‐INR	Per 1	3.28 (1.63–6.59)	0.001	Not selected	
D‐dimer	Per 5 μg/mL	1.10 (1.08–1.13)	< 0.001	1.09 (1.07–1.10)	< 0.001
ALT	Per 1 U/L	1.01 (1.00–1.02)	0.025	1.01 (1.00–1.02)	0.032
Anticoagulation status (+)	vs. (−)	3.50 (1.54, 7.97)	0.003	Not selected	
Sex (Female)	vs. male	0.88 (0.44–1.75)	0.71		
Lung cancer histology (Adenocarcinoma)	vs. others	0.89 (0.47–1.66)	0.71		
Medical history of myocardial infarction (+)	vs. (−)	1.92 (0.46–7.98)	0.37		
Comorbidity					
Hypertension (+)	vs. (−)	0.87 (0.47–1.63)	0.67		
COPD (+)	vs. (−)	1.84 (0.96–3.56)	0.07		
Diabetes (+)	vs. (−)	1.04 (0.48–2.25)	0.92		
Hyperlipidemia (+)	vs. (−)	0.99 (0.48–2.08)	0.98		
Atrial fibrillation (+)	vs. (−)	3.54 (1.49–8.41)	0.004	7.29 (2.72–19.52)	< 0.001

*Note:* Not selected: Variables not selected by stepwise method.

Abbreviations: ALT, alanine transaminase; ATE, arterial thromboembolism; CI, confidence interval; HR, hazard ratio; VTE, venous thromboembolism.

### 3.5 Incidence Rate of ATE According to VTE Development and EDO Administration

**FIGURE 4:**
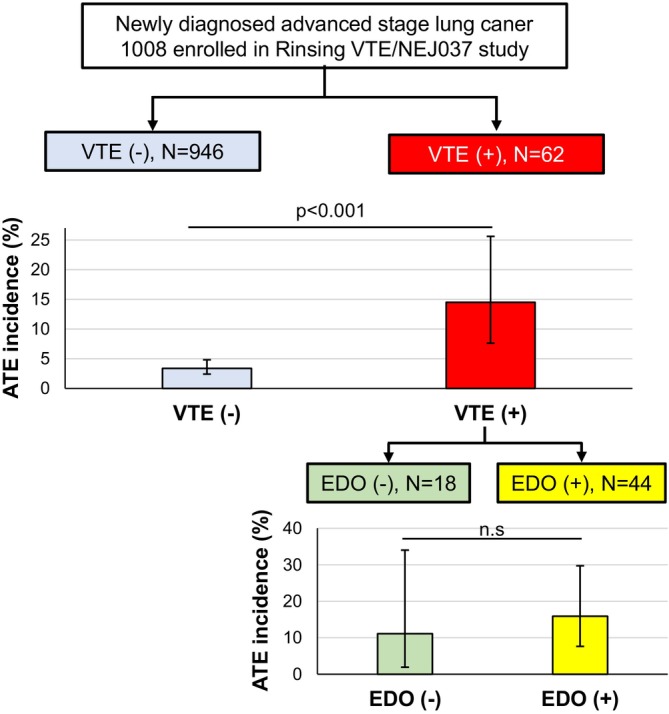
Incidence rate of ATE according to VTE development and EDO administration. ATE, arterial thromboembolism; VTE, venous thromboembolism; EDO, Edoxaban.

According to multivariate analysis, VTE was identified as a strong risk factor for the development of ATE. Therefore, we performed additional analyses for the incidence rate of ATE according to the development of VTE and the administration of EDO (Table 4). The incidence rate of ATE in VTE (+) patients was significantly higher than VTE (−) patients (14.5% vs. 3.4%, *p* < 0.001). In VTE (+) patients, the incidence rate of ATE was 15.9% (7/44) for the EDO (−) patients, compared with 11.1% (2/18) for the EDO (+) patients (*p* = 0.626). EDO administration did not contribute to a reduction of ATE in patients who developed VTE (Figure 4).

**TABLE 4:** Incidence rate of ATE according to VTE development and EDO administration with the Gray model.

All patients	VTE (+) patients *N* = 62	VTE (−) patients *N* = 946	All patients *N* = 1008
Number of ATE developed cases	9	32	41
Incidence rate of ATE (%) (95%CI)	14.5% (7.6–25.6)	3.4% (2.4–4.8)	4.1% (3.0–5.5)
VTE (−) vs. VTE (+)	*p* < 0.001		

EDO administration	EDO (+) patients *N* = 44	EDO (−) patients *N* = 18	
Number of ATE‐developed cases	7	2	
Incidence rate of ATE (%) (95%CI)	15.9% (7.6–29.7)	11.1% (1.9–34.0)	
EDO (+) vs. EDO (−)	*p* = 0.626		

Abbreviations: ATE, arterial thromboembolism; EDO, edoxaban; VTE, venous thromboembolism.

## 4 Discussion

The Rising‐VTE/NEJ037 study is the largest prospective cohort study to intensively screen for VTE from patients diagnosed with lung cancer over 2 years. We investigated the incidence rate and risk factors of ATE as secondary endpoints. To the best of our knowledge, there is no larger lung cancer‐specific prospective study to identify the risk factors of ATE in patients with lung cancer. In the present study, the incidence rate of ATE was 4.1% over a 2‐year follow‐up. Multivariate analysis suggested that VTE would be an independent risk factor for the development of ATE, and that intervention with DOACs for VTE did not contribute to the prevention of ATE.

According to a retrospective cohort study that investigated cancer‐associated ATE including multiple cancer types in Denmark [24], the authors reported that the cumulative incidence rate of ATE was 2.7%, 12 months after cancer diagnosis in lung cancer patients. In the present study, the cumulative incidence rate of ATE was higher since the follow‐up duration was 2 years. Since survival time for lung cancer patients is getting longer due to the use of immune checkpoint inhibitors and molecular targeted therapy, ATE events are expected to further increase in the future.

As highlighted in the Rising‐VTE/NEJ037, and as we experience in clinical practice, the most common ATE event was cerebral infarction. The following mechanisms can be contribute to the development of cancer‐associated stroke, (i) aseptic vegetations on mitral valve embolisms to brain through aorta, (ii) intracranial tumor compression over the middle cerebral artery (MCA) and causing occlusion, (iii) radiation vasculopathy causing multifocal stenoses of the internal carotid artery (ICA), (iv) deep venous thrombosis (DVT) causing paradoxical embolism in the setting of the atrial septal shunt (patent foramen oval; PFO) [25]. Therefore, patients who develop cancer‐associated stroke should be treated based on the mechanisms of each patient [26].

As we previously reported, EDO administration demonstrated incredible efficacy for VTE treatment and prevention, and the recurrence rate of VTE was 0% at both 6 months and 2 years after initiation of EDO [21]. However, there is only limited data to address the management of ATE in patients with cancer [22]. In particular, it was unclear whether DOCAs were effective in the prevention of cancer‐associated ATE. In the present analyses, there was no statistical difference seen between EDO (+) and EDO (−) groups for the incidence rate of ATE (EDO (+) 15.9% vs. EDO (−) 11.1%, for the EDO (+), *p* = 0.626). In other words, DOACs might be less effective in the prevention of ATE, including stroke (Figure 4). However, for patients with DVT and PFO, DOACs might be useful for the prevention of paradoxical embolism mechanistically. Recent meta‐analysis revealed that anticoagulant use was not associated with a reduction in ATE events compared to placebo or standard treatment [27], which was compatible with our present study. Further studies are needed to clarify the efficacy of DOACs for ATE. One ongoing randomized study to evaluate EDO by comparing low‐molecular weight heparin for acute ischemic stroke patients with active cancer [28, 29], might shed light on the use of DOACs for acute phase cancer‐associated ATE.

This study included several limitations. First, this study was designed to detect VTE incidence rates, the present analyses were just the secondary endpoints. The number of ATE events totaled only 41 cases in this cohort, which might be insufficient to perform multivariate analysis to identify the risk factors of ATE accurately. Future larger prospective studies should be conducted to investigate ATE as a primary end‐point. Second, ATE occurred mostly as a cerebral infarction, and atrial fibrillation was also identified as an independent risk factor in the present study. Moreover, the median age in the present study was 70, and this is relatively higher than in previous studies. Thus, asymptomatic paroxysmal atrial fibrillation could have developed in some older patients during the study period. We did not confirm whether the patients with atrial fibrillation were treated with appropriate anticoagulant therapy, nor did we collect data regarding the number of VTE (−) patients treated with anticoagulation for atrial fibrillation. Third, we did not consider the impact of anticancer drugs and smoking history. Anti‐VEGF antibodies, such as bevacizumab and ramucirumab, and immune checkpoint inhibitors are occasionally used for NSCLC treatment and can increase the risk of thromboembolism [30]. We did not collect the data for anticancer drug regimens in patients with lung cancer in detail and smoking history, but in general, anti‐VEGF antibodies are a contraindication for patients with thromboembolism. Finally, our study included only advanced‐stage lung cancer patients. The present findings might not be applied to other cancer types.

## 5 Conclusion

The incidence rate of ATE was 4.1% over a 2‐year follow‐up in patients with advanced lung cancer. Furthermore, VTE was an independent risk factor for ATE, and intervention with DOACs was less effective for the prevention of ATE in cases of advanced lung cancer with VTE.

## Author Contributions

**Naoki Furuya:** investigation (equal), writing – original draft (lead). **Yukari Tsubata:** conceptualization (lead), funding acquisition (lead), investigation (lead), methodology (lead), project administration (lead), resources (lead), supervision (lead), writing – review and editing (lead). **Takamasa Hotta:** investigation (equal), writing – review and editing (equal). **Toshihide Yokoyama:** investigation (equal), writing – review and editing (equal). **Masahiro Yamasaki:** investigation (equal), writing – review and editing (equal). **Nobuhisa Ishikawa:** investigation (equal), writing – review and editing (equal). **Kazunori Fujitaka:** investigation (equal), writing – review and editing (equal). **Tetsuya Kubota:** investigation (equal), writing – review and editing (equal). **Kunihiko Kobayashi:** investigation (equal), supervision (supporting), writing – review and editing (equal). **Takeshi Isobe:** conceptualization (lead), funding acquisition (lead), investigation (equal), methodology (lead), project administration (lead), resources (lead), supervision (lead), writing – review and editing (equal).

## Ethics Statement

This study was conducted in accordance with Helsinki Declaration and the Good Clinical Practice Guidelines. The study protocol was approved by the institutional review board in Shimane University (approval number: CRB6180008).

## Consent

Written informed consent was obtained from all patients before enrollment of this study.

## Conflicts of Interest

Naoki Furuya received personal fees from AstraZeneca K.K., Chugai Pharmaceutical, Nippon Boehringer Ingelheim Co. Ltd., Bristol‐Myers Squibb Company, Eli Lilly Japan K.K., MSD K.K., Pfizer Japan Inc., Taiho Pharmaceutical, and Novartis Pharma K.K. outside the submitted work. Yukari Tsubata received grant and personal fees from Daiichi Sankyo Co. Ltd. and AstraZeneca K.K. and personal fees from Chugai Pharmaceuticals Inc. outside the submitted work. Toshihide Yokoyama received personal fees from Eli Lilly Japan K.K., Merck Sharp & Dohme K.K., and Takeda Pharmaceutical outside the submitted work. Kazunori Fujitaka received personal fees from AstraZeneca K.K., Chugai Pharmaceutical Co. Ltd., Bristol‐Myers Squibb Company, MSD K.K., Pfizer Japan Inc., Daiichi Sankyo Co. Ltd., Eli Lilly Japan, and Nippon Boehringer Ingelheim Co. Ltd. outside the submitted work. Kunihiko Kobayashi received personal fees from AstraZeneca K.K. and Takeda Pharmaceutical outside the submitted work. Takeshi Isobe received grant and personal fees from Daiichi Sankyo Co. Ltd.; personal fees from AstraZeneca K.K., Pfizer Japan Inc., and Nippon Boehringer Ingelheim Co. Ltd.; and grants from Pearl Therapeutics Inc. and Janssen Pharmaceutical K.K. outside the submitted work. The remaining authors have nothing to disclose.

## Data Availability

The data of the external validation cohort from the Rising‐VTE study will be shared on reasonable request to the corresponding author.
